# Development of a Novel Gastrointestinal Endoscopic Robot Enabling Complete Remote Control of All Operations: Endoscopic Therapeutic Robot System (ETRS)

**DOI:** 10.1155/2019/6909547

**Published:** 2019-11-04

**Authors:** Keiichiro Kume, Nobuo Sakai, Toru Ueda

**Affiliations:** ^1^Third Department of Internal Medicine, School of Medicine, University of Occupational and Environmental Health, Japan, Kitakyushu, Japan; ^2^Department of Applied Science for Integrated System Engineering, Faculty of Engineering, Kyushu Institute of Technology, Japan

## Abstract

**Background and Objective:**

The master and slave transluminal endoscopic robot and other flexible endoscopy platforms are designed primarily for the remote control of forceps, with manipulation of the endoscope itself still dependent on conventional techniques. We have developed an endoscopic therapeutic robot system (ETRS) that provides complete remote control of all forceps and endoscope operations.

**Method:**

We carried out endoscopic submucosal dissection (ESD) in porcine stomachs using the ETRS. All procedures were completed with the endoscopist seated at the console the entire time.

**Results:**

Total en bloc resection was achieved in all 7 cases with no complications. The mean total procedure time was 36.14 ± 14.98 min, the mean size of the resected specimen was 3.39 ± 0.66 cm × 3.03 ± 0.63 cm, and the mean dissection time was 14.91 ± 8.61 min.

**Conclusion:**

We successfully used the ETRS to perform completely remote-controlled ESD in porcine stomachs.

## 1. Introduction

Flexible endoscopy platforms such as the master and slave transluminal endoscopic robot (MASTER) [1–3] and the EndoSAMURAI [4–6] have been designed primarily for the remote control of forceps, with manipulation of the flexible endoscope itself still dependent on conventional manual techniques. This dependence limits developments in this field, and accomplishing treatment with one endoscopist using intuitive manipulation is generally considered important. In our experience, a good sense of the movement of any type of forceps in the field of view visible on the monitor is limited to the area within a 10 mm radius of the tip of the endoscope. Remote control of the movement not only of the forceps but also of the endoscope itself is thus required to achieve extensive maneuverability beyond this limit. We started by developing a platform to remotely control movements of the endoscope itself, which we named the Endoscopic Operation Robot (EOR) [7–9]. The current third-generation EOR is equipped with two-way haptic feedback functions that provide haptic feedback (force sensation) via the master unit while transmitting a force equal to that applied by the operator on the master unit to the endoscope tip, and all scope operations can be performed one-handed [10, 11].

We have now developed a master-slave system capable of remotely operating three different endoscopic instruments (grasping forceps, knife forceps, and injection-needle catheters), and we have combined this with the improved EOR version 3 to create a novel gastrointestinal endoscopic robot in which all operations are completely remote-controlled: the endoscopic therapeutic robot system (ETRS). The performance of endoscopic submucosal dissection (ESD) in porcine stomachs using the ETRS is reported.

## 2. Methods

### 2.1. The Endoscopic Therapeutic Robot System (ETRS)

**Figure 1:**
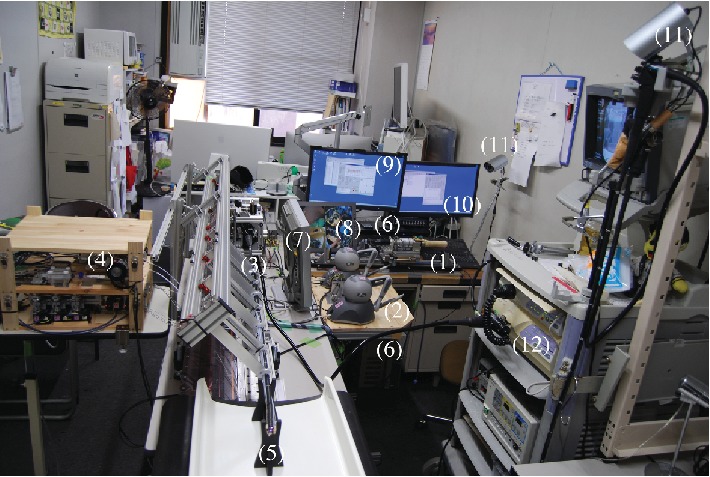
The endoscopic therapeutic robot system (ETRS) consisting of a master unit of an endoscope control system (1), a master unit of a forceps control system (2), a slave unit of an endoscope control system (3), a control unit of a forceps control system (4), the tip of the endoscope (5), two personal computers (PC) (6), an endoscope monitor (7), a robot operation monitor (8), a PC monitor of an endoscope control system (9), a PC monitor of a forceps control system (10), two robot operation cameras (11), and an endoscope system.

**Figure 2:**
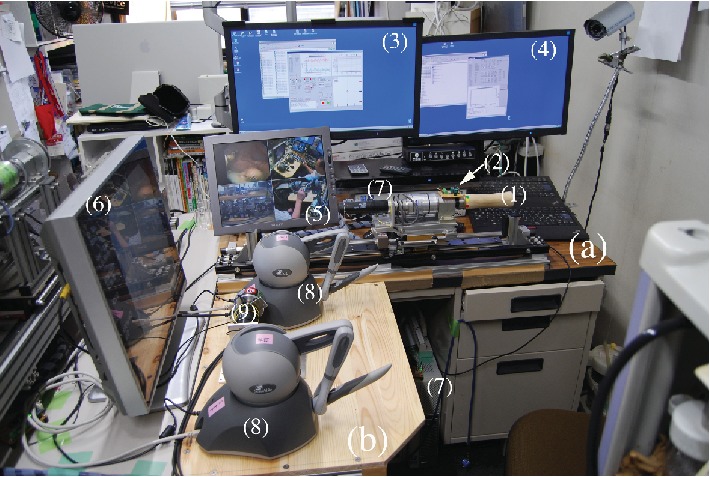
The L-shaped console consisting of the endoscope control system on the right (a) and the forceps control system in front (b): rotating handle (1), mini joystick (2), a PC monitor of an endoscope control system (3), a PC monitor of a forceps control system (4), a robot operation monitor (5), an endoscope monitor (6), two personal computers (PC) (7), two Geomagic Touch devices (8), and a master unit of the injection-needle catheter system (9).

**Figure 3:**
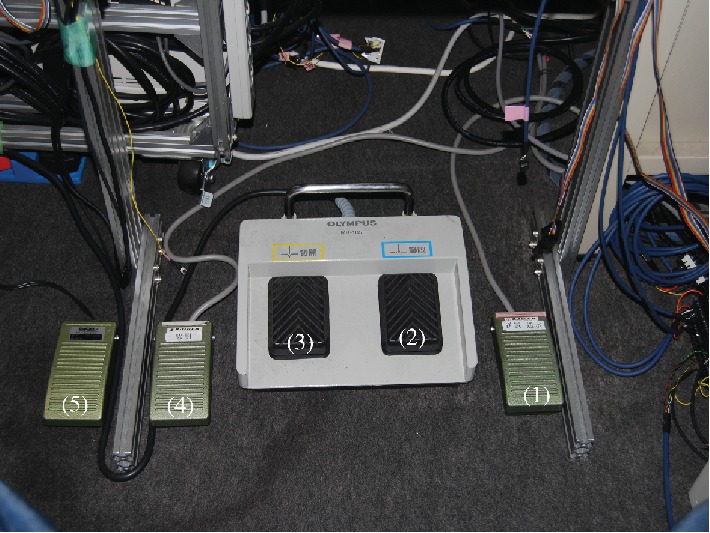
Five foot switches: controlling air and lens washing (two-stage switch) (1), coagulation (2), incision (3), suction (4), and water jet supply (5).

**Figure 4:**
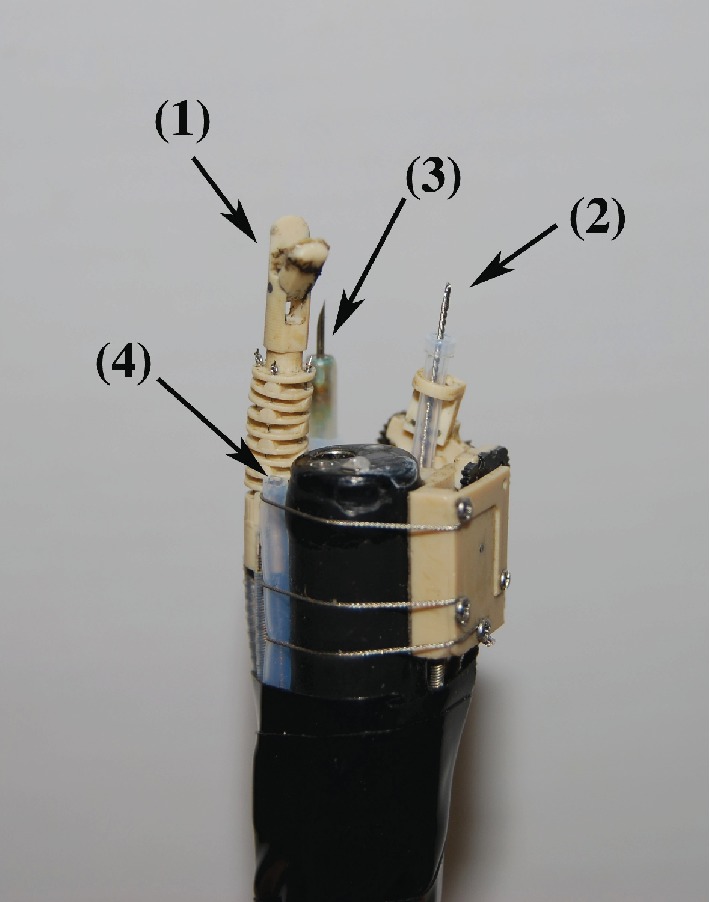
The tip of the endoscope is fitted with grasping forceps (1) and knife forceps with an attached drive unit (2), a puncture catheter (3), and a water jet supply tube (4).

The ETRS, a completely remote-controlled gastrointestinal endoscopic robot platform, is a novel master-slave robot that enables a single endoscopist to complete endoscopic treatment while seated at the console (Figure 1, video 1). The L-shaped console is designed to enable a single seated endoscopist to carry out all operations, with the forceps control system in front and the endoscope control system on the right (Figure 2). The forceps control system has the following construction. The rotatable grasping forceps and knife forceps can be manipulated in three dimensions using one Geomagic Touch unit (3D Systems Inc., Valencia, California, USA) for each forceps on the master unit, enabling their two-handed operation with two units. Each Geomagic Touch stylus has two buttons: on the left-hand stylus, pressing the front button manipulates the grasping forceps and pressing the rear button opens and closes them, while on the right-hand stylus, pressing the front button manipulates the knife forceps and pressing the rear button moves the tip of the knife in and out. The injection-needle catheter is operated by a master unit equipped with a haptic feedback function that we have developed, which enables puncture sensation to be monitored during its operation. Drug solution injection was controlled by a potentiometer. The endoscope control system used was the unmodified EOR version 3 master unit. As previously reported, the EOR version 3 is an original device designed for intuitive operation that enables all the elements of flexible endoscopy to be performed with one hand [10, 11]. It has five foot switches, which, from the right, are used to control air and lens washing (two-stage switch), coagulation, incision, suction, and water jet supply (Figure 3). The tip of the endoscope (GIF-XQ240, Olympus Medical systems Corp., Tokyo, Japan) is fitted with grasping forceps and knife forceps (Flex knife, Olympus) with an attached drive unit, a puncture catheter, and a water jet supply tube (Figure 4). The entire endoscope including the tip, where its diameter is widest, can be inserted inside an overtube (20 double type; inside diameter 20 mm, Top Corporation, Tokyo, Japan).

### 2.2. ESD Procedure (Video 2)

ESD in porcine stomachs using this ETRS was done by one experienced endoscopist. The conventional ESD was performed in a clinically established method (marking around the simulated lesion, submucosal local injection, cutting around the simulated lesion, submucosal dissection, and removing the resected simulated lesion). All procedures were completed with the endoscopist seated at the console the entire time.

### 2.3. Outcomes

The following parameters were recorded for all ESD procedures: all procedure time, dissection time, size of the resected specimen, dissection speed (dissection time per specimen size (min/cm^2^) and specimen size per dissection time (cm^2^/min)), and adverse events.

The size of the resected specimen (cm^2^) was calculated using the formula *πab*/4, respectively, where *a* is the length of the long axis and *b* is the length of the short axis.

## 3. Results

A total of 7 cases of ESD performed with ETRS were successfully completed with *en bloc* resection. There were no complications. Details of findings are presented in Table 1.

The mean total procedure time was 36.14 ± 14.98 min, the mean size of the resected specimen was 3.39 ± 0.66 cm × 3.03 ± 0.63 cm and 8.33 ± 3.06 cm^2^, the mean dissection time was 14.91 ± 8.61 min, and the mean dissection speed is 1.88 ± 1.05 min/cm^2^ and 0.67 ± 0.32 cm^2^/min.

## 4. Discussion

We have been working on the development of a robot system that will enable sophisticated, high-precision diagnosis and treatment irrespective of the operator's skill, by moving away from the current situation in which diagnosis and treatment are performed using a hand-held endoscope and making all the operations involved remote-controlled. For this to happen, it will be important to switch from the present skills of expert endoscopists, who carry out treatment by endoscope and forceps manipulation alone, to the “intuitive operability” provided by our master unit. The ETRS consists of (1) a forceps control system and (2) an endoscope control system, and we focused mainly on developing the forceps control system using the Geomagic Touch device. The Geomagic Touch is a master unit with a haptic feedback function, but because of technical limitations, we did not make use of this function in our system; instead, we considered that stylus-controlled operation to simulate holding the forceps in both hands would be more intuitive. We also considered that haptic feedback to provide puncture sensation was essential in order to puncture the gastric wall with injection forceps, and we developed our own master-slave unit for this purpose. The EOR version 3 was used as the endoscope control system. The main role of the EOR version 3 is to access the area requiring treatment. In addition to providing access in the broad sense of reaching the treatment region, in a narrower sense, it provides the access required for precise movements to treatment sites that cannot be dealt with forceps manipulation alone in the limited operating space of the gastrointestinal tract, including access operations such as movements adjacent to the lesion while maintaining an appropriate distance and controlling the angle of approach. The endoscope fitted with the forceps system unit is sufficiently narrow that it can be operated smoothly within an overtube in current clinical use. Hence, it is clinically usable in terms of size.

Flexible endoscopy platforms are classified into two groups with remote forceps operation and assisted endoscope operation (manipulation of flexible endoscopes themselves). The MASTER [1–3], EndoSAMURAI [4–6], 3D-printed overtube system [12], and 2.6 mm articulating devices [13] are included in the former. EOR and the robotic-assisted flexible endoscope (RAFE) [14] are included in the latter. ETRS is the only platform to combine both of these operations.

MASTER is the only robot to have been used to carry out actual clinical dissection of ESD. However, the only procedure for which the MASTER has yet been used is dissection of the submucosa, with marking and surrounding incision being performed with a regular endoscope, and it was necessary to switch among three different endoscopes [1–3, 7]. On the other hand, it is unnecessary for ETRS to switch to other endoscopes. The EndoSAMURAI is a platform with two arms fitted to the tip of an endoscope that includes forceps channels in addition to the endoscope itself and its two arms, meaning that, if necessary, three different sets of forceps can be used interchangeably at the same time [4–7]. However, it is impossible for ETRS to change to other forceps. As for the 3D-printed overtube system, forceps are passed through the side channels of the overtube sleeve and are guided by the flexible arms of an adaptor located at the end of the overtube [12]. In brief, exchange of the forceps is possible, like the EndoSAMURAI. The 2.6 mm articulating devices include grasping forceps and electric knives that have an articulating part for up/down and right/left directions. These devices are manually driven by a wire system connected to the handles [13], whereas the ETRS is driven by computer control. RAFE is similar to EOR of our previous robot system [14]. RAFE is more compact than EOR.

In the reports of ESD using RAFE [14], the 3D-printed overtube system [12], and 2.6 mm articulating devices [13], dissection time and specimen size were shown. We compared the mean dissection speed of these systems and ETRS. When mean dissection speed was not described in each manuscript, we calculated it using the data reported in each manuscript. The mean dissection speed of ETRS was the shortest of these systems (Table 2).

The ETRS enables a single endoscopist to complete endoscopic treatment while seated at a console. However, questions still remain as to its reliability, safety, and durability, and we intend to continue to make improvements in preparation for its clinical application while also developing new gastrointestinal endoscopic treatment methods that use the simultaneous manipulation of multiple forceps and the complete remote control of operations further.

In conclusion, we have developed a novel master-slave robot, the ETRS, which enables a single endoscopist to complete endoscopic treatment while seated at a console. We successfully used the ETRS to perform completely remote-controlled ESD in porcine stomachs.

## Figures and Tables

**Table 1:** Outcomes of 7 endoscopic submucosal dissection procedures.

No. of case	Specimen size		Total procedure time (min)	Dissection time (min)	Dissection speed	
	cm × cm	cm2			Dissection time per specimen size (min/cm2)	Specimen size per dissection time (cm2/min)
1	2.5 × 2.0	3.92	24.4	5.8	1.48	0.68
2	2.5 × 2.0	4.32	39.1	16.5	3.82	0.26
3	4.0 × 3.5	11.00	58.1	29.7	2.70	0.37
4	4.2 × 3.5	11.55	53.5	22.2	1.92	0.52
5	3.5 × 3.5	9.62	23.7	11.4	1.19	0.84
6	3.5 × 3.0	8.25	20.1	6.8	0.82	1.21
7	3.5 × 3.5	9.62	34.1	11.7	1.22	0.82

**Table 2:** Comparison between dissection speed of ETRS and other systems.

Name of system	Mean specimen size	Mean dissection speed	
		Mean dissection time per specimen size (min/cm2)	Mean specimen size per dissection time (cm2/min)
RAFE	5.35	2.62	—
3D-printed overtube system	5.29	—	0.45
2.6 mm articulating devices	4.24	2.12	0.62
ETRS	8.32	1.88	0.67

RAFE: the robotic-assisted flexible endoscope; ETRS: the endoscopic therapeutic robot system (our system).

## Data Availability

The data of this study were included in Table 1. These data were carefully checked.

## Supplementary Materials

Supplementary Materials Video 1: the functions and method of operation of the ETRS. Video 2: the performance of ESD in a porcine stomach using the ETRS. The video displays four divided images; these show the operation, as captured by the 2 robot operation cameras: console operation in close-up (right lower), console operation from distance (right upper), commentary of operation (left lower), and the endoscope monitor (left upper).
