# Erratum to “Development of a Novel Gastrointestinal Endoscopic Robot Enabling Complete Remote Control of All Operations: Endoscopic Therapeutic Robot System (ETRS)”

**DOI:** 10.1155/2020/6173978

**Published:** 2020-04-27

**Authors:** Keiichiro Kume, Nobuo Sakai, Toru Ueda

**Affiliations:** ^1^Third Department of Internal Medicine, School of Medicine, University of Occupational and Environmental Health, Japan, Kitakyushu, Japan; ^2^Department of Applied Science for Integrated System Engineering, Faculty of Engineering, Kyushu Institute of Technology, Japan

In the article titled “Development of a Novel Gastrointestinal Endoscopic Robot Enabling Complete Remote Control of All Operations: Endoscopic Therapeutic Robot System (ETRS)” [1], there was an error in Supplementary Materials (available here), whereby the second supplementary video was omitted. This error occurred during the production process. The correct supplementary materials are as follows.

## Supplementary Materials

Supplementary Materials Video 1: the functions and method of operation of the ETRS. Video 2: the performance of ESD in a porcine stomach using the ETRS. The video displays four divided images; these show the operation, as captured by the 2 robot operation cameras: console operation in close-up (right lower), console operation from distance (right upper), commentary of operation (left lower), and the endoscope monitor (left upper).
